# Real-World Snapshot of Dietary Patterns in Subjects Living with Chronic Kidney Disease

**DOI:** 10.3390/nu17243864

**Published:** 2025-12-11

**Authors:** Mariana Di Lorenzo, Maria Serena Lonardo, Mariastella Di Lauro, Martina Chiurazzi, Anna Fiorenza de Giovanni di Santa Severina, Marcella Capuano, Bruna Guida, Rossella Trio, Daniela Pacella, Andrea Memoli, Simona Esposito

**Affiliations:** 1 Physiology Nutrition Unit, Department of Clinical Medicine and Surgery, University of Naples Federico II, Via Sergio Pansini 5, 80131 Naples, Italy; marianadilorenzo.mdl@gmail.com (M.D.L.); mslonardo91@gmail.com (M.S.L.); martinachiurazzi88@gmail.com (M.C.); fiorenzadegiovanni@gmail.com (A.F.d.G.d.S.S.); marcellacapuano@gmail.com (M.C.); bguida@unina.it (B.G.); rostrio@unina.it (R.T.); 2 Department of Public Health, University of Naples Federico II, Via Sergio Pansini 5, 80131 Naples, Italy; daniela.pacella@unina.it; 3 Nephrology Unit, Pellegrini Hospital, Via Portamedina alla Pignasecca, 41, 80134 Naples, Italy; andrea_memoli@hotmail.it; 4 Department of Medicine and Surgery, LUM University, Strada Statale 100 km 18, Casamassima, 70010 Bari, Italy; simonaesposito.248@gmail.com

**Keywords:** chronic kidney disease, dietary habits, ultra-processed food, food processing, diet quality

## Abstract

**Background/Objectives**: Chronic kidney disease (CKD) represents a major global public health challenge. Diet plays a central role in CKD management, with guidelines emphasizing individualized intake of energy, macronutrients, and micronutrients in combination with medical treatment. In recent years, increasing attention has been directed toward diet quality and the degree of food processing, particularly the consumption of ultra-processed foods (UPFs), which have been linked to adverse metabolic and renal outcomes. However, limited data are available on the real-life dietary patterns of individuals with CKD who have not yet received structured nutritional counseling. This study aims to describe the dietary habits of adults with CKD compared to healthy controls, in order to better understand nutritional challenges and identify potential targets for dietary intervention in CKD management. **Methods**: 73 subjects (46.6% M) were enrolled; they attended the Outpatients Clinic of the I.P. “Diet Therapy in transplantation, renal failure and chronic pathology”, University of Naples Federico II. Subjects were divided into two groups based on the presence/absence of CKD, established on the basis of a glomerular filtration rate (eGFR) < 60 mL/min/1.73 m^2^. Each participant was evaluated for biochemical parameters, anthropometric measurements, body composition, and dietary assessment. **Results**: CKD group showed a lower caloric intake compared to Control Group. In particular, lipid intake was significantly higher in Control Group whereas carbohydrates intake was higher in CKD Group. No difference was found between the two groups regarding daily protein intake. Dietary sodium and salt intake was found to be lower in CKD Group compared to Control Group and the latter showed a lower omega-6/omega-3 ratio. Interestingly, the consumption of UPF was higher in Control Group compared to CKD Group. **Conclusions**: This study offers a snapshot of the dietary habits of a cohort from Southern Italy CKD stage 3–5 patients, showing that even in the absence of specific nutritional guidance, individuals were able to implement small lifestyle changes such as UPF and salt intake reduction. However, critical nutritional imbalances in CKD patients show the limits of self-managed diets, highlighting the need for structured nutritional support.

## 1. Introduction

Chronic kidney disease (CKD) is a major public health concern worldwide, affecting roughly 11% of the adult population [1]. It is closely linked to cardiovascular disease in a bidirectional manner [2] and the growing prevalence of hypertension and type 2 diabetes—which are key risk factors for CKD [3,4]—is expected to further increase the global burden of this chronic condition [5]. Without proper management, CKD can gradually progress to end-stage renal disease, requiring dialysis or kidney transplantation [6]. Clinical management involves pharmacological treatment alongside lifestyle modifications such as smoking cessation [7], reducing alcohol consumption [8], increasing physical activity [9], and adopting healthier dietary habits [10,11]. Specifically, diet plays a central role in both the progression of CKD and the patient’s overall well-being [12]. In this context, clinical guidelines provide specific dietary recommendations, emphasizing the importance not only of the quality of diet but, recently, increasing attention has been paid to the degree of food processing in the management of subjects with CKD [13]. Although dietary guidelines are often, if not exclusively, used by health professionals, public campaigns promoting healthy lifestyles are becoming increasingly common, and information is being shared more with the general population [14]. For this reason, there is a growing need to move from nutrient-specific recommendations toward a more holistic focus on overall dietary patterns and diet quality, considering other aspects of the food, such as the degree of processing [13,15,16]. In this context, the Nova classification was developed to categorize foods according to their level of processing, under the premise that the extent and purpose of processing may exert distinct effects on human health beyond those attributable to nutritional composition alone [17]. Ultra-processed foods (UPFs) are defined as industrial formulations predominantly or entirely composed of substances derived from foods, or synthesized from food constituents, frequently enriched with flavorings, colorants, emulsifiers, and other cosmetic additives, and containing minimal or no intact whole foods (e.g., carbonated beverages, processed meats, packaged sweet or savory snacks) [16]. They are characteristically high in added saturated and trans fats, added sugars, salt, and inorganic phosphate additives while being low in dietary fiber, vitamins, and antioxidants [18,19] and typically exhibit high energy density, limited satiety power, and broad accessibility, factors that collectively promote excessive intake [20]. Surprisingly, even in southern Italy, traditionally considered the cradle of the Mediterranean diet, there is an increasing shift towards more Western dietary patterns that are rich in UPFs [21] and there is a growing body of evidence that has linked their consumption to adverse metabolic [22,23] and renal outcomes both in the general population and in individuals with CKD [24,25].

Nevertheless, little is known about the frequency with which these foods are consumed by individuals with CKD in real life settings, particularly those who are not yet receiving structured nutritional support. Most existing dietary research in CKD has focused on prescribed dietary interventions, often overlooking the spontaneous or habitual food choices made by individuals outside of a clinical setting. Therefore, this study aims to address a relevant knowledge gap: the lack of data on UPF consumption among CKD subjects in everyday life in the absence of formal dietary counseling. As we hypothesized that CKD individuals without tailored dietary counseling may follow suboptimal dietary patterns, the stated aim is to provide a descriptive snapshot of the dietary habits of adults with CKD compared with healthy controls in order to contribute to a more nuanced understanding of nutritional challenges in CKD and identify significant differences and potential areas for nutritional intervention in the management of CKD.

## 2. Materials and Methods

### 2.1. Study Design and Data Collection

This single-center case–control study was approved by the Ethical Committee of the Federico II University Medical School of Naples on 18 July 2018 (Project identification code 181/18) and was conducted according to the guidelines laid down in the Declaration of Helsinki. Written informed consent was obtained from all participants.

Between March 2023 and January 2024, a total of 73 subjects (46.6% M) attending the Outpatients Clinic of the I.P. “Diet Therapy in transplantation, renal failure and chronic pathology”, University of Naples Federico II, were enrolled. Participants were stratified into two groups based on the presence/absence of CKD established on the basis of a glomerular filtration rate (eGFR) < 60 mL/min/1.73 m^2^:

- Control Group: subjects without CKD.
- CKD Group: subjects living with CKD (stage 3–5) following free-living diet and not yet in conservative therapy.

Participants were selected as follows:

- Age between 18 and 65 years old.
- Body Mass Index (BMI) ≥ 18.5 kg/m^2^.
- Free living diet (habitual diet without any strict dietary control or supervision).

Participants with eating disorders (e.g., bulimia, anorexia), recent changes in drug therapy, food allergies or intolerances, warfarin use, those who were bedridden or had gastrointestinal malabsorption syndromes or on protein-free diet, gastrointestinal symptoms (diarrhea, nausea, vomiting, early satiety), cancer, dementia, depression, neurological disorders or infections were excluded from the study. Pregnant and lactating women were also excluded.

Data such as age, gender, education level (categorized as secondary school or below, high school, university), place of residence (metropolitan area or small and medium-sized cities), smoking habits, and the time elapsed from initial diagnosis (in months), was collected. Self-reported physical activity level (sedentary, medium, heavy) was also recorded.

History of diabetes, hypertension and hypercholesterolemia was defined by current use of pharmacological treatment.

### 2.2. Anthropometric Measurements and Body Composition Analysis

For this evaluation, all subjects had to be without shoes and in light clothes [26]. Body weight and height were determined using a calibrated balance beam scale and a stadiometer (Seca 711; Seca Hamburg, Germany) and BMI was subsequently calculated. As previously reported, waist circumference (WC) was assessed using an un-stretched tape measure, midway between the lower edge of the rib cage and the iliac crest, according to the National Institutes of Health (NIH) protocols [27].

In order to assess body composition, bioelectrical impedance analysis (BIA) was conducted using the Akern device (BIA 101 BIVA^®^ PRO AKERN srl, Florence, Italy). The exam was carried out according to ESPEN guidelines [28] and parameters as fat-free mass (FFM), fat mass (FM), total body water (TBW), extracellular water (ECW) expressed in %, and phase angle (Φ) were collected.

### 2.3. Biochemical and Clinical Parameters

Overnight fasting venous blood samples were collected from all enrolled subjects and blood parameters such as glucose, insulin, total cholesterol (Tot-C), HDL cholesterol (HDL-C), LDL cholesterol (LDL-C), triglycerides (TG), uric acid, creatinine, blood urea, serum electrolytes (potassium, phosphorus, calcium), serum hemoglobin and ferritin were evaluated by using standard analytic laboratory methods. Briefly, TG/HDL-C ratio, a practical alternative to HOMA-IR for identifying subjects with insulin resistance (IR), was calculated [29]. The estimated eGFR was evaluated using CKD-EPI formula [30]. In addition, all participants were investigated for hypertension according to the 2021 ESC guidelines using an aneroid sphygmomanometer and the procedure was repeated 3 times at 1 min apart [31].

### 2.4. Dietary Assessment

Data on daily caloric, macronutrient intake and salt consumption were obtained using the FFQ BLOCK questionnaire, specifically validated for the population with CKD [32] that includes 100 items. More specifically, in this questionnaire, patients were asked how many servings (as defined in the questionnaire) of each considered food they consumed during the period of interest. From the data obtained by this questionnaire and by comparing them with specific tables showing the nutrient composition of each food, the nutrient and energy intake was calculated. In particular, we used the CREA food composition tables [33].

The NOVA Food Frequency Questionnaire (NFFQ), a validated questionnaire aimed to evaluate the food consumption of Italian adults according to NOVA group classification [34], was administered to each recruited subject by a qualified nutritionist during a face-to-face interview. Participants were instructed to respond based on their dietary habits during a typical month in the past 12 months. Each answer corresponded to one of ten frequency categories: (1) “never or less than once a month”, (2) “one to three times per month”, (3) “once a week”, (4) “twice a week”, (5) “three times per week”, (6) “four times per week”, (7) “five times per week”, (8) “six times per week”, (9) “every day”, and (10) “if every day, how many times per day?”. In addition to consumption frequency, participants reported their usual portion size by selecting one of six options, ranging from 0.5 to 3 portions. The food items included in the questionnaire were organized into nine categories: (1) fruit and nuts, (2) vegetables and legumes, (3) cereals and tubers, (4) meat and fish, (5) milk, dairy products, and eggs, (6) oils, fats, and seasonings, (7) sweets and sweeteners, (8) beverages, and (9) other. An additional table was provided for reporting commonly consumed items not covered in the previous sections. As previously reported [23], the NOVA classification was used to categorize each food item into one of the following categories according to the extent and purpose of food processing: non-processed or minimally processed foods (MPFs), processed culinary ingredients (PCIs), processed foods (PFs) and ultra-processed foods (UPFs). Specifically, when processing NFFQ data, PCIs were grouped with PFs, as these products are not intended to be consumed alone but are usually used to prepare other foods [35] and the g/day consumed of each groups and the percentage they represented with respect to the total amount of food eaten were determined in order to obtain a weight-ratio (%) that is more appropriate than an energy ratio since it better accounts for non-nutritional factors related to food processing [36].

In addition, adherence to the Mediterranean Diet (MD) was assessed by administering the questionnaire PREvención con DIetaMEDiterránea (PREDIMED). This validated questionnaire consists of 14 items concerning the consumption of typical MD foods and, by assigning a score of 0 or 1 for each item, the PREDIMED score was calculated as follows: 0–5, low adherence; 6–9, medium adherence; ≥10, high adherence [37].

### 2.5. Statistical Analysis

Categorical variables are presented as absolute numbers and percentages (%). The Kolmogorov–Smirnov test was used to assess the normality of data distribution. The Levene test was used to evaluate the equality of variance and Welch’s *t*-test was used in cases where variances differed. Normally distributed variables are expressed as the mean ± standard deviation (SD), while non-normally distributed variables are reported as the median and interquartile range (IQR). For comparisons between independent groups, Student’s *t*-test or non-parametric test, Mann–Whitney, and Chi-square were used. Comparisons between independent groups were conducted using one-way ANOVA. Bonferroni correction was used for post hoc pairwise comparisons. Non-normally distributed variables were expressed as the median and interquartile range and a nonparametric test of multiple comparison of Krustal-Wallis was executed. Correlation was calculated using Spearman’s correlation coefficient. For further confirming our results, we used a propensity score matching to balance the two groups by several potential confounding variables. Optimal matching was achieved using nearest neighbor matching with probit link and a caliper of 0.8, with a 1:1 ratio without replacement. The matching was conducted by the following covariates: age, sex BMI, hypertension, diabetes and dyslipidemia. Statistical analysis was performed using SPSS version 20.0 (SPSS Inc., Chicago, IL, USA), with statistical significance set at *p* < 0.05.

## 3. Results

Table 1 and Table 2 depict the baseline demographic features, anthropometric measures, body composition as well as biochemical and clinical parameters of the study population [n = 73, 46.6% males, age of 55.0 (95% CI: 45.0–72.0)]. No differences between the two groups in terms of anthropometric characteristics were observed. However, a difference in body composition parameters was found. Specifically, CKD Group showed significantly lower values of FM (25.9 ± 9.9% vs. 32.3 ± 7.2%, *p* = 0.02), and Phase Angle (5.7 ± 0.9 vs. 6.5 ± 1.1, *p* = 0.02) compared to Control Group. Still, in CKD Group, a significant increase in FFM% (74.0 ± 9.9% vs. 67.6 ± 7.1%, *p* = 0.02), TBW% (54.9 ± 7.2% vs. 50.0 ± 5.4%, *p* = 0.02) and ECW% (47.4 ± 4.8% vs. 44.4 ± 4.4%, *p* = 0.04) values was detected. Data obtained also indicate a significantly higher prevalence of hypertension and dyslipidemia in CKD Group compared to Control Group (Table 1).

In addition, Tot-C (161.4 ± 38.0 vs. 184.1 ± 42.4, *p* = 0.02) and HDL-C levels (48.3 ± 10.6 vs. 55.7 ± 14.4, *p* = 0.02) were lower in CKD Group compared to Control Group. Furthermore, ferritin [82.8 (95% CI: 41.0–168.0) vs. 53.3 (95% CI: 37.8–83.8), *p* = 0.03] and uric acid levels [5.1 (95% CI: 4.4–6.5) vs. 4.8 (95% CI: 3.9–5.8), *p* = 0.02] were found to be higher in CKD Group compared to Control Group (Table 2). No significant differences were highlighted for the other biochemical and clinical parameters considered (Table 2).

As shown in Table 3, CKD Group had a significantly lower total caloric intake compared to Control Group [2036 (95% CI: 1745.4–2398.0) vs. 3234 (95% CI: 2539.2–3746.0), *p* = 0.000]. Specifically, lipid intake was significantly higher in Control Group [29.0 (95% CI: 25.5–31.5) vs. 37.0 (95% CI: 31–44.7), *p* = 0.000] whereas carbohydrate intake was significantly higher in CKD Group [52.0 (95% CI: 49.0–56.0) vs. 45.0 (95% CI: 37.5–49.7), *p* = 0.000]. No difference was found between the two groups regarding daily protein intake (Table 3). Dietary sodium [1724.9 (95% CI: 1286.7–2287.2) vs. 2291.5 (95% CI: 1411.0–30.92.5), *p* = 0.03] and salt intake [4.3 (95% CI: 3.2–5.7) vs. 5.7 (95% CI: 3.5–7.7), *p* = 0.03] was found to be significantly lower in CKD Group than Control Group and the latter showed a significantly lower omega-6 to omega-3 ratio [5.2 (95% CI: 4.4–6.6) vs. 4.1 (95% CI: 3.6–4.8), *p* = 0.000] (Table 3). Furthermore, no differences were found in adherence to the Mediterranean diet, which was found to be average in both groups as the PREDIMED score was [6.0 (95% CI: 5–8) in CKD Group vs. 6.0 (95% CI: 5.2–7) in Control Group] (Table 3).

The daily consumption of unprocessed or minimally processed foods (MPF) and processed foods (PF + PCI) was found to be comparable between the two groups (Supplementary Tables S1 and S2). In detail, from the total of MPF consumption, the intake of fresh legumes [21.4 (95% CI: 0.0–21.4) vs. 0.0 (95% CI: 0.0–11.2), *p* = 0.007] was significantly higher in CKD Group than Control Group and no difference was found regarding the other MPFs considered (Table S1). In addition, from the total of PF (PF + PCI) consumption, the intake of concentrated sweeteners and sugar based products [15 (95% CI: 8.9–26.6) vs. 8.6 (95% CI: 0.0–16.9), *p* = 0.008] was significantly higher in CKD Group compared Control Group, while oil intake [30.0 (95% CI: 20.0–40.0) vs. 40.0 (95% CI: 30.0–47.5), *p* = 0.01] and pizza artisanal intake [50 (95% CI: 35.0–50.0) vs. 55 (95% CI: 50.0–50.0), *p* = 0.01] were significantly lower in CKD Group than Control Group (Supplementary Table S2). The consumption of ultra-processed foods (UPF) was significantly lower in CKD Group (*p* = 0.01) (Table S3). In detail, in terms of daily consumption, in Control Group the percentage of UPF consumption was 18.2% (95% CI: 12.9–26.6) and in CKD Group it was 11.9% (95% CI: 8.8–15.9). From the total of UPF consumed, the intake of chocolate [1.4 (95% CI: 0.0–6.4) vs. 8.6 (95% CI: 0.0–18.7), *p* = 0.007], cereals and bars [ 0.0 (95% CI: 0.0–0.0) vs. 3.6 (95% CI: 0.0–24.1), *p* = 0.001], spreadable creams [0.0 (95% CI: 0.0–1.5) vs. 1.5 (95% CI: 0.0–10.7) *p* = 0.009], ultra-processed dairy [0.0 (95% CI: 0.0–12.5) vs. 26.7 (95% CI: 0.0–53.6), *p* = 0.005] and fish sticks [0.0 (95% CI: 0.0–10.0) vs. 10.0 (95% CI: 0.0–14.3), *p* = 0.007] were found to be lower in CKD Group (Table S3). No differences were found in the daily consumption of the other UPFs considered (Supplementary Table S3). In addition, our results revealed a difference in UPF consumption not only between the Control and CKD group, but also across individual CKD stages groups. In particular, a statistically significant difference was observed between CKD stage 3 group and the Control Group (% UPF 10.8 (8.7–16.4) vs. 18.2 (12.9–26.6), *p* = 0.009; g UPF 202.3 (126.4–292.1) vs. 285.5 (211.8–380.1), *p* = 0.024) (Supplementary Table S4). Furthermore, a significant negative correlation was observed between UPF consumption (% of tot food and g/die) and PREDIMED SCORE (r = 0.4, *p* = 0.001, UPF% of tot food; r = 0.3, *p* = 0.003, UPF g/die). In the end, after applying the propensity score, our results were confirmed, except for LDL-C, HDL-C, uric acid, ferritin, chocolate, oil, artisanal pizza and concentrated sweeteners and sugar based products (Supplementary Tables S5–S11).

## 4. Discussion

Several epidemiological studies have well documented that a high intake of ultra-processed foods (UPFs) increases the risk of developing chronic kidney disease (CKD) and accelerates its progression [24,38,39]. Indeed, UPFs, due to their high sodium, phosphorus and potassium content, can exacerbate metabolic disorders common in CKD, such as hypertension, hyperphosphatemia and hyperkaliemia. In addition, additives and low fiber content can contribute to systemic inflammation and intestinal dysbiosis, further accelerating renal function decline [40]. Beyond inflammation, oxidative stress represents another key pathophysiological mechanism linking UPF consumption to CKD progression. UPFs are typically high in pro-oxidant compounds, advanced glycation end-products (AGEs), and synthetic additives, while being poor in antioxidants and bioactive nutrients. This nutritional profile can increase reactive oxygen species (ROS) production, impair endogenous antioxidant defenses, and exacerbate oxidative damage to renal tissues. Evidence indicates that oxidative stress plays a major role in mediating cardiovascular and metabolic complications in CKD, contributing to endothelial dysfunction, tubular injury, and accelerated loss of renal function. The recent literature underscores the double burden of UPF consumption and CKD, highlighting how these foods may amplify oxidative and inflammatory pathways that are already heightened in kidney disease [25,41].

However, despite this evidence, little is currently known about UPF consumption in people who have already been diagnosed with CKD. In this view, this study offers novel insights into the spontaneous dietary habits of adults living with CKD, compared to control subjects, with a particular focus on UPF consumption.

One of the main surprising takeaways is the lower consumption of UPFs in the CKD group compared to the Control Group and particularly in CKD stage 3 group. It can be hypothesized that people with poor kidney function, especially in the early stages of the disease, may be more aware of their eating habits and limit their consumption of UPFs in order to manage the progression of the disease and any related complications. Therefore, lower UPF consumption among CKD patients may reflect a cautious approach to diet in this study population. It is plausible to assume that this cautiousness may be influenced, at least in part, by public health campaigns aimed at raising awareness of the risks associated with UPFs. These initiatives, often targeting the general population or people living with chronic diseases such as CKD, may indirectly contribute to more conservative food choices in this subject group.

Although there is no direct evidence on the effect of public health campaigns specifically targeting UPF consumption in the CKD population, awareness-raising initiatives do exist. For example, Padial et al. developed targeted infographics to educate people with CKD about UPFs, how to identify them, and how to make healthier, kidney-friendly choices [42]. Additionally, while recent reviews do not describe active awareness campaigns, they do promote increased awareness among both the general population and healthcare professionals about the risks associated with UPF consumption in CKD [40,43]. Considering that UPFs represent a major source of hidden salt in the modern diet [16], another noteworthy finding, consistent with their reduced intake observed in our vulnerable population, was a lower dietary sodium and dietary salt intake compared to healthy population. This is a positive result, given that excessive sodium intake is a key risk factor for hypertension, which can further worsen kidney function in individuals with CKD. Indeed, sodium reduction is generally recommended in the CKD management to help control blood pressure and reduce the risk of cardiovascular events [13].

Interestingly, although CKD Group had a lower dietary salt intake, their higher potassium levels (a marker of impaired kidney function) suggest that the management of other electrolytes, such as potassium, may also require attention. Therefore, a balanced approach to mineral intake remains essential in dietary interventions for the chronic kidney disease population.

Despite the reduced consumption of UPFs, the overall nutritional quality of the diet observed in individuals with CKD still reveals notable areas of concern. Specifically, this group exhibited a lower total caloric intake alongside a disproportionately high percentage of energy derived from carbohydrates, particularly from processed concentrated sweeteners and sugar-based products.

The increased carbohydrates intake detected in CKD Group may reflect a combination of dietary counseling, appetite-related changes, metabolic adaptations and taste alterations common in CKD individuals [44]. Dysgeusia in subjects with CKD, particularly in advanced stages, is very common and can result from several CKD-related factors, including the accumulation of uremic toxins, dry mouth, medication use and micronutrient deficiencies which alter the perception of both taste and smell [44]. This alteration in taste can significantly impact food choices, contributing to an increase in carbohydrate consumption, particularly simple sugars or starchy foods because the perception of sweet taste tends to remain relatively preserved, while the perception of salty and bitter tastes is frequently impaired [45,46]. Hence, it is conceivable that a direct link between taste alterations reduced salt tolerance as well as consumption of sodium-rich UPFs and, consequently, there was a compensatory increase in the consumption of carbohydrates. However, from a metabolic perspective, higher carbohydrates consumption, particularly sweet or refined carbohydrates, could pose additional challenges for CKD management as such intake may exacerbate kidney disease and contribute to insulin resistance, dyslipidaemia, and worsening cardiovascular risk, which are already elevated in this population [47]. Another possible explanation may relate to the lower fat intake observed in the CKD Group, which could drive a compensatory increase in carbohydrate percentage to maintain overall caloric intake. This macronutrient redistribution may be unintentional but reflects the nutritional imbalance often seen in patients with progressive CKD.

The higher omega-6/omega-3 ratio observed in the patient group, despite their lower caloric intake, may be explained by a relatively poor dietary quality. Regarding the intake of fats, it is important to consider not only the quantity but also the quality of fats consumed. This imbalance plays a critical role in modulating inflammation and may significantly influence CKD progression. Indeed, a high *n*-6/*n*-3 ratio, typical of Western diets, has been associated with increased production of pro-inflammatory eicosanoids that exacerbate systemic inflammation, a hallmark of CKD pathophysiology. [48].

Surprisingly, no significant difference in daily protein intake was found between CKD and non-CKD subjects. Although this unexpected finding may reflect inadequate nutritional awareness and knowledge among these individuals, such an interpretation remains purely speculative. An alternative explanation is that some individuals with CKD may intentionally limit their protein intake based on informal or non-evidence-based advice, leading to inconsistent dietary behaviors that obscure clear differences between groups. Food knowledge, defined as the ability to understand, interpret, and apply dietary information, represents a cornerstone of CKD management. It enables individuals to understand how nutrients interact with impaired kidney function, thereby supporting informed and sustainable food choices that promote long-term health and well-being [49].

The lower caloric intake observed in Group 1 may reflect either intentional dietary restriction, after diagnosis, as often advised for individuals with CKD, or involuntary appetite loss, a common symptom in this population [50]. Indeed, anorexia in CKD is a multifactorial and progressive condition, arising from a vicious cycle of physiological disruptions. As renal function declines, uremic toxins accumulate, triggering systemic inflammation, oxidative stress and alterations in central appetite regulation. These factors act synergistically with taste disturbances, gastrointestinal dysfunctions and hormonal dysregulation (notably ghrelin and leptin), all of which contribute to marked reductions in appetite and food intake [51].

This reduced nutrient intake in turn can lead to malnutrition and protein-energy wasting (PEW), a state of pathological loss of body protein and energy reserves, frequently observed in CKD and strongly associated with poor clinical outcomes and reduced quality of life. The paradoxical finding of higher FFM in CKD Group compared with controls may not necessarily indicate a healthier body composition; rather, it likely was due to a relative expansion in extracellular water compartment (ECW) due to fluid retention and hyperhydration, which are common in CKD. Such fluid shifts can mask true muscle wasting and therefore explain the apparent increase in FFM. Furthermore, this observation is also supported by the concomitant lower PhA indicating that subjects belonging to this group have a lower body cell mass than the Control Group, probably due to the shift in water from the intracellular to the extracellular compartment or a higher degree of tissue inflammation [52,53]. All these findings reinforce the need for personalized nutrition counseling and education programs aimed at improving individuals’ understanding of dietary choices, food labeling, and portion sizes in order to achieve a balanced diet with optimal macronutrient intake, adequate to preserve lean mass and prevent malnutrition, but sufficiently restrictive to minimize the workload on the kidneys and uremic symptoms, thereby improving clinical outcomes in CKD.

Furthermore, these data could contribute to the development of specific dietary guidelines for patients with CKD, with a focus on reducing UPF intake as a key component of CKD management. By promoting more conscious and sustainable eating behaviors, these strategies have the potential to improve clinical outcomes and overall quality of life for people with CKD.

### Limitations

Although this study provides valuable insights, several limitations must be acknowledged. The study’s cross-sectional design means that we cannot establish causality between UPF intake and CKD progression. Additionally, the sample size is relatively small and the study was conducted at a single center, which may limit the generalizability of the findings. Larger, multi-center studies with long-term follow-ups are needed to establish definitive links between UPF consumption and long-term outcomes in CKD population.

Third, dietary data were collected using a food frequency questionnaire (FFQ), which is subject to recall bias and may not fully capture true intake. Additionally, inflammatory and oxidative stress biomarkers, key indicators for interpreting nutritional status and CKD-related metabolic alterations, were not assessed. The study also did not account for informal sources of nutritional information, which may influence dietary behaviors such as the reduction in salt intake independent of formal counseling.

## 5. Conclusions

This study offers a snapshot of the dietary habits of a cohort from Southern Italy diagnosed with CKD, highlighting associations between CKD status and certain dietary patterns, such as lower UPF and salt intake. While these observations may suggest a degree of dietary awareness, it is important to note that causality cannot be inferred, and the findings may be influenced by factors such as nutritional literacy, symptom-driven behaviors, or informal dietary advice underscoring both the potential and the limitations of spontaneous dietary self-management in this population. Nonetheless, structured nutritional intervention delivered by a specialized team remains the cornerstone for effectively supporting kidney health but also for preserving nutritional status, preventing metabolic complications, and improving quality of life in people living with CKD.

## Figures and Tables

**Table 1:** Demographic, anthropometric and body composition characteristics of the study population.

	All Patients n = 73	Control Group n = 28	CKD Group n = 45
Age, Years	55.0 (45.0–72.0)	50.5 (40.2–58.0)	60.0 (60.5–65.5)
Male, n (%)	34 (46.6%)	9 (32.1%)	25 (55.6%)
Education level, n (%)			
Secondary school or below	29 (40.8%)	9 (33.3%)	20 (45.5%)
High school	18 (25.4%)	9 (33.3%)	9 (20.5%)
University	3 (4.2%)	0 (0.0%)	3 (6.8%)
Place of residence, n (%)			
Metropolis	98 (56%)	19 (67.9%)	21 (46.7%)
Small and medium-sized cities	40 (54.8%)	9 (32.1%)	24 (53.3%)
Physical activity level, n (%)			
Sedentary	76 (56.7%)	22 (78.6%)	34 (75.6%)
Medium	14 (19.2%)	4 (14.3%)	10 (22.2%)
Heavy	3 (4.1%)	2 (7.1%)	1 (2.2%)
Current smoking n (%)	16 (22.9%)	6 (24.0%)	10 (22.2%)
CKD stage, n (%)	Stage 3–5: n. 45 (61.6%)		Stage 3–5: n. 45 (100%)
Diabetes	17 (23.9%)	3 (11.5%)	14 (31.1%)
Dyslipidemia	31 (43.7%)	7 (26.9%)	24 (53.3%) *
Hypertension	49 (69.0%)	11 (42.3%)	38 (84.4%) °
BMI, kg/m^2^	30.4 ± 6.4	31.7 ± 4.2	29.6 ± 7.4
WC, cm	98 (91.5–105.5)	98.2 (91.2–104)	98.0 (91.5–106.0)
FFM, %	55.9 ± 8.3	66.5 ± 6.8	72.1 ± 9.7 *
FM, %	30.0 ± 9.1	33.4 ± 6.9	27.9 ± 9.7 *
TBW, %	51.9 ± 6.5	49.4 ± 5.0	53.6 ± 6.8 *
ECW, %	46 ± 4.5	44.2 ± 4.7	47.2 ± 4.0 *
Phase Angle, Φ	6.0 ± 0.9	6.5 ± 1.1	5.8 ± 0.8*

Continuous variables are expressed as mean ± SD. Categorical variables are expressed as numbers and percentages. Abbreviations are: BMI, Body Mass Index; WC, Waist Circumference; FFM, Fat-Free Mass; FM, Fat Mass; ECW, Extracellular water; TBW, Total Body Water; CKD, Chronic kidney disease. * *p* < 0.05 vs. Control Group, ° *p* < 0.001 vs. Control Group.

**Table 2:** Biochemical and clinical characteristics of the study population.

	All Patients n = 73	Control Group n = 28	CKD Group n = 45
Glucose, mg/dL	93.3 ± 15.7	89.9 ± 11.4	95.5 ± 17.7
TG/HDL ratio	2.6 ± 1.5	2.2 ± 15.7	2.9 ± 15.7
Tot-C, mg/dL	170.5 ± 41.1	184.1 ± 42.4	161.4 ± 38.0 *
LDL-C, mg/dL	95.7 ± 34.1	102.9 ± 30.6	85.1 ± 37.1
HDL-C, mg/dL	51.4 ± 12.8	55.7 ± 14.4	48.3 ± 10.6 *
TG, mg/dL	120.8 ± 52.2	113.8 ± 50.5	125.4 ± 53.4
Uric acid, mg/dL	5.6 (4.4–6.5)	4.8 (3.9–5.8)	5.1 (4.4–6.5) *
Creatinine, mg/dL	1.7 (0.9–2.5)	0.8 (0.7–0.9)	2.3 (1.8–3.2) °
Blood urea, mg/dL	71.9 ± 42.9	35.2 ± 6.6	92.3 ± 40.7 °
eGFR (CKD-EPI)	54.1 ± 34.5	92.7 ± 17.1	29.5 ±13.7 °
Potassium, mg/dL	4.7 ± 0.5	4.4 ± 0.3	4.8 ± 0.5 °
Phosphorus, mg/dL	3.9 ± 0.6	3.7 ± 0.5	3.9 ± 0.7
Calcium, mg/dL	9.4 ± 0.6	9.4 ± 0.6	9.4 ± 0.5
Hemoglobin, g/dL	12.9 ± 1.8	13.3 ± 1.6	12.7 ± 1.9
Albumin, g/dL	4.1 ± 0.4	4.1 ± 0.5	4.1 ± 0.4
Ferritin, ng/mL	82.8 (41.0–168.0)	53.3 (37.8–83.8)	109.5 (48.7–184.2) *
SBP, mmHg	120 (120.0–131.5)	120 (111.2–130.0)	120 (120–133.7)
DBP, mmHg	80 (75.0–80.0)	80 (80.0–80.0)	80 (76.2–80)

Data are expressed as mean ± SD or median and interquartile range (IQR). Abbreviations are: Tot-C, Tot Cholesterol; LDL-C, Low-Density Lipoprotein-cholesterol; HDL-C, High-Density Lipoprotein-cholesterol; TG, Triglyceride; SBP, Systolic Blood Pressure; DBP, Diastolic Blood Pressure; TG/HDL ratio, triglyceride/high-density lipoprotein ratio; EGFR, Estimated Glomerular Filtration Rate. * p < 0.05 vs. Control Group 0, ° *p* < 0.001 vs. Control Group.

**Table 3:** Dietary characteristics of the study population.

	All Patients n = 73	Control Group n = 28	CKD Group n = 45
Kcal/day	2368.7 (1868.8–3234.0)	3234 (2539.2–3746.0)	2036 (1745.4–2398.0) °
Intake CHO, % of tot food	51.0 (43.8–55.0)	45.0 (37.5–49.7)	52.0 (49.0–56.0) °
Intake LIP, % of tot food	31.0 (27.0–37.4)	37.0 (31–44.7)	29.0 (25.5–31.5) °
Intake PRO, % of tot food	18.0 (15.2–20.5)	8.0 (15.0–21.0)	17.0 (15.5–20.0)
PREDIMED SCORE	6.0 (5.0–7.0)	6.0 (5.2–7)	6.0 (5–8)
Sodium intake, mg/day	1910 (1335.0–2500.0)	2291.5 (1411.0–30.92.5)	1724.9 (1286.7–2287.2) *
Salt intake, g/day	4.7 (3.3–6.3)	5.7 (3.5–7.7)	4.3 (3.2–5.7) *
Omega 6/Omega 3	4.7 (4.0–6.1)	4.1 (3.6–4.8)	5.2 (4.4–6.6) °

Data are expressed as mean ± SD or median and interquartile range (IQR). Abbreviations are: CHO, carbohydrate; LIP, lipid; PRO, protein. * *p* < 0.05 vs. Group 0, ° *p* < 0.001 vs. Group 0.

## Data Availability

The original contributions presented in this study are included in the article/Supplementary Materials. Further inquiries can be directed to the corresponding author.

## Supplementary Materials

The following supporting information can be downloaded at: https://www.mdpi.com/article/10.3390/nu17243864/s1, Table S1: Intake of Minimally processed foods within the study population; Table S2: Intake of Processed foods within the study population; Table S3: Intake of Ultra-pocessed food within the study population; Table S4: Variation of dietary intake with advancing CKD in the study population; Table S5: Demographic, anthropometric and body composition characteristics of the study population; Table S6: Biochemical and clinical characteristics of the study population; Table S7: Dietary characteristics of the study population; Table S8: Intake of Minimally processed foods within the study population; Table S9: Intake of Processed foods within the study population; Table S10: Intake of Ultra-pocessed food within the study population; Table S11: Variation of dietary intake with advancing CKD in the study population.

## Abbreviations

The following abbreviations are used in this manuscript:

BIA	Bioelectrical Impedance Analysis
BMI	Body Mass Index
CKD	Chronic Kidney Disease
CREA	Consiglio Per La Ricerca In Agricoltura E l’Analisi Dell’economia Agraria
DBAP	Diastolic Blood Arterial Pressure
DM	Diabetes Mellitus
eGFR	Estimated Glomerular Filtration Rate
FFM	Fat-Free Mass
FFQ	Food Frequency Questionnaire
FM	Fat Mass
Hba1c	Glycated Hemoglobin
HDL-C	HDL-Cholesterol
KDOQI	Kidney Disease Outcomes Quality Initiative
LDL-C	LDL-Cholesterol
NIH	National Institutes Of Health
PA	Phase Angle
PEW	Protein-Energy Wasting
SBAP	Systolic Blood Arterial Pressure
SD	Standard Deviation
TBW	Total Body Water
TC	Total Cholesterol
TG	Triglycerides
WC	Waist Circumference
CHO	Carbohydrate
LIP	Lipid
PRO	Protein
TG/HDL ratio	Triglyceride/high-density lipoprotein ratio
ECW	Extracellular water
