# Supplementary material for: Real-World Snapshot of Dietary Patterns in Subjects Living with Chronic Kidney Disease
Source: Nutrients. 2025 Dec 11;17(24):3864. doi: 10.3390/nu17243864 (PMC12735972; doi:10.3390/nu17243864)
Supplement: Supplementary file 1 [file nutrients-17-03864-s001.zip › nutrients-3987597-supplementary.pdf]

Supplementary Table S1: Intake of Minimally processed foods within the study population

	**All patients** n=73	**Control Group** n=28	**CKD Group** n=45
**MPF g/day**	945.8±285.7	951.6 ±251.9	942.1 ±239.9
**% of tot food**	57.4±12.9	55.6 ±12.2	58.6 ±13.3
**MPF** ***s' Subgroups***			
Fruits **g/day**	300.0 (64.3-300.0)	175.0 (16.6-300.0)	300.0 (96.4-300.0)
**100% fruit or vegetables juices g/day**	0.0 (0.0-0.0)	0.0 (0.0-0.0)	0.0 (0.0-7.0)
**Dried fruit g/day**	0.0 (0.0-0.0)	0.0 (0.0-0.0)	0.0 (0.0-0.0)
**Vegetables g/day**	200.0 (198.6-415.4)	251.4 (98.6-406.0)	234.3 (208.0-422.8)
**Dried legumes g/day**	7.1 (0.0-7.6)	2.5 (0.0-8.4)	7.1 (2.5-7.1)
**Fresh legumes g/day**	15.0 (0.0-21.4)	0.0 (0.0-11.2)	21.4 (0.0-21.4)*
**Meet g/day**	64.0 (35.5-85.8)	64.1 (42.0-112.4)	57.1 (29.0-85.7)
**Fish g/day**	32.0 (15.0-60.5)	24.7 (0.0-60.7)	32.1 (21.4-60.5)
**Milk g/day**	12.2 (0.0-137.5)	125.0 (0.0-187.5)	0.0 (0.0-125.0)
**Tubers g/day**	28.6 (14.3-57.1)	28.5 (0.0-28.6)	28.6 (14.3-57.1)
**Pasta and whole cereal grains g/day**	79.9 (50.7-117.1)	67.5 (34.4-127.1)	79.9 (61.4-113.4)
**Eggs g/day**	14.3 (7.1-21.4)	14.2 (8.1-26.8)	14.3 (7.1-19.6)
**Plain yogurt g/day**	0.0 (0.0-0.0)	0.0 (0.0-29.9)	0.0 (0.0-29.9)
**Coffee g/day**	60.0 (17.1-90.0)	60.0 (17.1-90.0)	60.0 (23.6-90.0)

Data are expressed as mean±SD or median and interquartile range (IQR). Abbreviations are: MPF, Non-Processed or Minimally Processed Food. *$p < 0.05$ vs Control Group.

Supplementary Table S2: Intake of Processed foods within the study population

	All patients<br>n=73	Control Group<br>n=28	CKD Group<br>n=45
**PF (PF+PCI) g/day**	453.6±239.2	408.6± 168.7	481.6±272.1
**% of tot food****	26.9 ±9.8	24.8 ±8.1	28.2±10.7
**PF *s' Subgroups***			
**Oil g/day**	40.0 (20.0-40.0)	40.0 (30.0-47.5)	30.0 (20.0-40.0)*
**Olives g/day**	3.5 (0.0-10.0)	3.5 (0.0-10.0)	5.0 (2.0-8.5)
**Fats and dairy fats g/day**	0.0 (0.0-1.0)	0.0 (0.0-1.0)	1.0 (0.0-1.2)
**Concetrated sweeteners and sugar based products g/day**	11.4 (5.0-24.3)	8.6 (0.0-16.9)	15 (8.9-26.6)*
**Bread Fresh g/day**	100.0 (50.0-200.0)	75 (44.6-150.0)	100 (50.5-200.0)
**Pizza artisanal g/day**	50 (35.0-50.0)	55 (50.0-50.0)	50 (35.0-50.0)*
**Cheese g/day**	42.2 (23.2-65.1)	44.0 (19.3-68.2)	40.8 (27.1-65.1)
**Processed meat g/day**	14.3 (7.1-21.4)	14.3 (7.1-26.4)	14.3 (2.5-21.2)
**Canned Fish g/day**	0.0 (0.0-0.0)	0.0 (0.0-0.0)	0.0 (0.0-0.0)
**Smoked fish g/day**	7.1 (5.0-14.3)	7.1 (7.0-14.3)	7.1 (2.9-14.3)
**Canned Legumes g/day**	18.2 (0.0-42.8)	18.2 (0.0-42.9)	21.4 (0.0-37.5)
**Canned Vegetables g/day**	21.4 (0.0-42.8)	21.4 (0.0-38.5)	21.4 (15.0-42.8)
**Fruit juices g/day**	0.0 (0.0-0.0)	0.0 (0.0-0.0)	0.0 (0.0-0.0)
**Syrup-preserved fruit g/day**	0.0 (0.0-0.0)	0.0 (0.0-0.0)	0.0 (0.0-0.0)
**Dried fruit g/day**	0.0 (0.0-0.0)	0.0 (0.0-0.0)	0.0 (0.0-0.0)
**Added tree nuts g/day**	0.0 (0.0-0.7)	0.0 (0.0-2.2)	0.0 (0.0-2.2)
**Polenta g/day**	0.0 (0.0-0.0)	0.0 (0.0-0.0)	0.0 (0.0-0.0)
**Savory pies g/day**	0.0 (0.0-0.0)	0.0 (0.0-0.0)	0.0 (0.0-0.0)
**Gnocchi g/day**	0.0 (0.0-0.0)	0.0 (0.0-0.0)	0.0 (0.0-0.0)
**Artisanal biscuits and cakes g/day**	10.0 (0.0-11.5)	0.0 (0.0-13.2)	10.0 (0.0.11.5)
**Wine g/day**	0.0 (0.0-17.8)	0.0 (0.0-16.5)	0.0 (0.0-31.2)
**Beer g/day**	0.0 (0.0-33.0)	0.0 (0.0-27.5)	0.0 (0.0-40.1)
**Homemade sauces and gravies g/day**	5.0 (0.0-7.1)	0.0 (0.0-6.6)	5.0 (0.0-7.1)

Data are expressed as mean±SD or median and interquartile range (IQR). Abbreviations are: PF, Processed Food; PCI, Processed Culinary Ingredient. **p<0.05* vs Control Group .

Supplementary Table S3. Intake of Ultra-pocessed food within the study population

	**All patients** n=73	**Control Group** n=28	**CKD Group** n=45
**UPF g/day**	221.6 (154.1-296.9)	285.5 (211.8-380.1)	201.4 (125.1-277.1)*
**% of tot food**	13.4 (9.9-19.4)	18.2 (12.9-26.6)	11.9 (8.8-15.9)*
UPFs' Subgroups			
**Soft Drinks g/day**	0.00 (0-94.1)	30.8 (0-94.3)	0.00 (0-70.6)
**Alcoholics g/day**	0.0 (0.0-0.0)	0.0 (0.0-0.0)	0.0 (0.0-0.0)
**Packaged breads g/day**	0.0 (0.0-14.3)	5.0 (0-21.4)	0.0 (0.0-12.6)
**Buns g/day**	12.0 (3.0-30.0)	12.8 (0.0-30)	8.6 (5.3-21.4)
**Sweet and savoury snacks g/day**	14.3 (4.6-41.8)	19.9 (4.5 -56.4)	11.4 (4.6-31.4)
**Biscuits g/day**	8.6 (1.8-25.7)	12.8 (0.7-27.7)	8.6 (1.8-25.7)
**Ice-cream g/day**	0.0 (0.0-4.7)	0.0 (0.0-0.0)	0.0 (0.0-8.5)
**Chocolate g/day**	3.0 (0.0-10.3)	8.6 (0.0-18.7)	1.4 (0.0-6.4)*
**Chips and French fries, g/day**	0.0 (0.0-15.0)	0.0 (0.0-26.3)	0.0 (0-15.0.0)
**Sausages and würstel g/day**	14.3 (0.5-15.0)	0.0 (0-14.3)	14.3 (0-17.5)
**Nuggets and sticks g/day**	0.0 (0-14.3)	0.0 (0-14.3)	0.0 (0-14.3)
**Fish sticks g/day**	0.0 (0.0-14.3)	10.0 (0.0-14.3)	0.0 (0.0-10.0)*
**Cereals and bars g/day**	10.0 (0.0-14.3)	3.6 (0.0-24.1)	0.0 (0.0-0.0)*
**Ultraprocessed dairy g/day**	0.0 (0.0-35.7)	26.7 (0.0-53.6)	0.0 (0.0-12.5)*
**Pre-packaged pizza, sandwich and savory pies g/day**	0.0 (0.0-0.0)	0.0 (0.0-0.0)	0.0 (0.0-8.0)
**Ready to eat sauces and gravies g/day**	1.8 (0.0-5.9)	1.8 (0.0-5.0)	1.8 (0.0-5.0)
**Ready to eat pasta gnocchi dishes g/day**	0.0 (0.0-0.0)	0.0 (0.0-0.0)	0.0 (0.0-7.5)
**Spreadable creams g/day**	0.0 (0.0-0.0)	0.0 (0.0-1.5)	1.5 (0.0-10.7)*
**Meat substitutes g/day**	0.0 (0.0-0.0)	0.0 (0.0-0.0)	0.0 (0.0-0.0)
**Pre-packaged vegetables g/day**	0.0 (0.0-0.0)	0.0 (0.0-0.0)	0.0 (0.0-0.0)
**Instant noodles or soups g/day**	0.0 (0.0-0.0)	0.0 (0.0-0.0)	0.0 (0.0-0.0)
**Candies g/day**	0.0 (0.0-3.4)	0.0 (0.0-1.2)	0.0 (0.0-5.4)
**Protein drinks g/day**	0.0 (0.0-0.0)	0.0 (0.0-0.0)	0.0 (0.0-0.0)
**Margarine g/day**	0.0 (0.0-0.0)	0.0 (0.0-0.0)	0.0 (0.0-0.0)

Data are expressed as mean±SD or median and interquartile range (IQR). Abbreviations are: UPF, Ultra-Processed Food.
*$p < 0.05$ vs Control Group .

Supplementary Table S4: Variation of dietary intake with advancing CKD in the study population

	Control Group n=28	Stage 3 Group n=22	Stage 4 Group n=13	Stage 5 Group n=10
**MPF g/day**	951.6±351.9	993.8 ±248.1	849.1 ±224.8	949.3 ±226.6
**% of tot food**	55.6±12.2	58.6 ±13.3	56.6 ±15.8	61.1 ±10.4
**PF(PF+PCI)g/day**	408.6 ±168.7	517.3 ±270.5	462.5 ±316.4	427.9 ±225.8
**% of tot food**	24.7 ±8.1	29.0 ±10.2	27.7 ±11.3	27.1 ±11.7
**UPF g/day**	285.5 (211.8-380.1)	202.3 (126.4-292.1)*	159.4 (125.1-259.4)	218.3(91.1-270.8)
**% of tot food**	18.2 (12.9-26.6)	10.8 (8.7-16.4)*	11.8 (8.6-16.1)	11.9(8.1-15.8)

Data are expressed as mean±SD or median and interquartile range (IQR). Abbreviations are: MPF, Non-Processed or Minimally Processed Food; PF, Processed Food; PCI, Processed Culinary Ingredien; UPF, Ultra-Processed Food. *$p < 0.05$* vs Control Group

Supplementary Table S5: Demographic, anthropometric and body composition characteristics of the study population

	All patients n=40	Control Group n=20	CKD Group n=20
**Age, Years**	54.3±11.1	52± 9.2	56.6±12.5
**Male, n (%)**	19 (47.5%)	7 (35.0%)	12 (60.0 %)
**Education level, n (%)**			
**Secondary school or below**	14 (35.9%)	7 (36.8 %)	7 (35.0%)
**High school**	11 (28.2%)	6 (31.6 %)	5 (25.0%)
**University**	2 (5.1%)	0 (0.0 %)	2 (10.0%)
**Place of residence, n (%)**			
**Metropolis**	22 (55.0%)	13 (65.0%)	9 (45.0%)
**Small and medium-sized cities**	18 (45.0%)	7 (35.0%)	11 (55.0%)
**Physical activity level, n (%)**			
**Sedentary**	29 (72.2%)	22 (78.6%)	13 (65.0%)
**Medium**	8 (20.0%)	4 (14.3%)	6 (30.0%)
**Heavy**	3 (7.5%)	2 (7.1%)	1 (5.0%)
**Current smoking n (%)**	9 (22.5 %)	4 (22.2 %)	5 (25.0 %)
**CKD stage, n (%)**	Stage 3-5: n. 20 (50.0.6 %)		Stage 3-5: n. 20 (100 %)
**Diabetes**	8 (20.0 %)	3 (15 %)	5 (25.0 %)
**Dyslipidemia**	16 (40.0 %)	6 (30.0 %)	10 (50.0 %)
**Hypertension**	27 (67.5 %)	11 (55.0 %)	16 (80.0 %)
**BMI, kg/m2**	29.9±6.9	30.9± 4.7	28.7 ± 8.6
**WC, cm**	99.0 ± 15.1	99.2±13.0	98.9 ±17.3
**FFM, %**	70.8±9.1	67.6±7.1	74.0±9.9*
**FM, %**	29.1±9.1	32.3±7.2	25.9±9.9*
**TBW, %**	52.4±6.7	50.0±5.4	54.9±7.2*
**ECW, %**	45.9± 4.8	44.4±4.4	47.4±4.8*
**Phase Angle, Φ**	6.1±1.1	6.5±1.1	5.7±0.9*

Continuous variables are expressed as mean±SD. Categorical variables are expressed as numbers and percentages.Abbreviations are: BW, Body Weight; BMI, Body Mass Index; WC, Waist Circumference; FFM, Fat-Free

Mass; FM, Fat Mass; ECW, Extracellular water ; TBW, Total Body Water; CKD, Chronic kidney disease. *$p<0.05$ vs Control Group.

Table S6: Biochemical and clinical characteristics of the study population

	**All patients** n=40	**Control Group** n=20	**CKD Group** n=20
**Glucose, mg/dL**	92.1± 12.9	90.6 ± 11.2	93.6 ± 14.8
**TG/HDL ratio**	1.9 (1.3-3.4)	1.8 (1.1-3.2)	1.9 (1.3-4.1)
**Tot-C, mg/dL**	167.5±45.6	183.4±46	148.9±38.4*
**LDL-C, mg/dL**	91.9±40.3	103.2 ± 33.8	85.1± 37.1*
**HDL-C, mg/dL**	51.9±14.4	55.8 ± 16.0	46.9 ± 10.4
**TG, mg/dL**	114.0±58.2	113.0 ± 50.9	115.2± 67.7
**Uric acid, mg/dL**	5.7± 1.5	5.3± 1.3	6.1±1.7
**Creatinine, mg/dL**	1.9 ±1.5	0.8 ±0.2	3.0 ±1.4°
**Blood urea, mg/dL**	69.2±44.1	35.2±7.2	99.9±40.7°
**eGFR (CKD-EPI)**	59.9±36.6	93.5±14.4	26.3 ±12.9°
**Potassium, mg/dL**	4.7±0.5	4.4±0.4	4.9±0.6°
**Phosphorus, mg/dL**	3.8±0.7	3.6±0.5	3.9±0.7
**Calcium, mg/dL**	9.3±0.7	9.2±0.5	9.4±0.5
**Hemoglobin, g/dL**	12.6±1.9	13.4±1.6	12.0±2.1*
**Albumin, g/dL**	4.1±0.5	4.1±0.6	4.1±0.4
**Ferritin, ng/mL**	114.8± 51.3	86.2± 88.5	138.1±89.5
**SBP, mmHg**	127.7 ±12.8	126.5± 14.8	129.0 ± 10.6
**DBP, mmHg**	78.8 ± 6.7	79.2± 5.9	78.3±7.6

Data are expressed as mean±SD or median and interquartile range (IQR).

Abbreviations are: Tot-C, Tot Cholesterol; LDL-C, Low-Density Lipoprotein-cholesterol; HDL-C, High-Density Lipoprotein-cholesterol; TG, Triglyceride; SBP, Systolic Blood Pressure; DBP, Diastolic Blood Pressure; TG/HDL ratio, ,triglyceride/high-density lipoprotein ratio; EGFR, Estimated Glomerular Filtration Rate. *$p<0.05$ vs Control Group 0, °$p<0.001$ vs Control Group

Table S7: Dietary characteristics of the study population

	All patients n=40	Control Group n=20	CKD Group n=20
**Kcal/day**	2368.7 (1868.8-3234.0)	3284.3±1119.0	1936.5±354.5°
**Intake CHO,% of tot food**	51.0 (43.8-55.0)	45.3±9.4	51.1±7.5*
**Intake LIP,% of tot food**	31.0 (27.0-37.4)	36.0±9.6	28.6 6.1*
**Intake PRO,% of tot food**	18.0 (15.2-20.5)	18.0 (15.0-21.5)	17.0 (15.5-21.0)
**PREDIMED SCORE**	6.0 (5.0-7.0)	6.0 (5.0-7.0)	6.0 (6.0-7.0)
**Sodium intake, mg/day**	1910 (1335.0-2500.0)	2547.5±1031.6	1632.8±755.8*
**Salt intake, g/day**	4.7 (3.3-6.3)	6.4± 2.6	4.1± 4.9*
**Omega6/Omega 3**	4.7 (4.0-6.1)	4.4±1.6	5.9±1.9°

Data are expressed as mean±SD or median and interquartile range (IQR). Abbreviations are: CHO, carbohydrate; LIP, lipid; PRO, protein. **p <0.05* vs Goup 0, *°p <0.001* vs Group 0

Supplementary Table S8: Intake of Minimally processed foods within the study population

	All patients n=40	Control Group n=20	CKD Group n=40
**MPF g/day**	914.6±307.0	940.7 ±382.3	888.6 ±214.3
**% of tot food**	55.9±14.1	54.1 ±13.3	57.6 ±14.9
**MPF *s' Subgroups***			
***Fruits* g/day**	250.0 (64.3-300.0)	250.0 (15.0-300.0)	225.0 (91.1-412.5)
**100% fruit or vegetables juices g/day**	0.0 (0.0-0.0)	0.0 (0.0-0.0)	0.0 (0.0-10.5)
**Dried fruit g/day**	3.0 (0.0-0.0)	3.0 (0.0-14.9)	1.5 (0.0-8.6)
**Vegetables g/day**	222.8 (198.6-415.4)	257.1 (204.1-417.1)	222.8 (112.8-274.3)
**Dried legumes g/day**	7.1 (0.0-7.6)	6.0 (0.0-7.8)	7.1 (5.0-12.3)
**Fresh legumes g/day**	15.0 (0.0-21.4)	0.0 (0.0-15.0)	21.4 (16.5-42.8)*
**Meet g/day**	57.1 (35.5-85.8)	57.1 (42.0-101.8)	42.8 (28.7-80.3)
**Fish g/day**	32.1 (15.0-60.5)	24.7 (3.7-48.2)	42.8 (21.4-62.2)
**Milk g/day**	12.2 (0.0-137.5)	68.5 (0.0-187.5)	6.2 (0.0-125.0)
**Tubers g/day**	28.6 (14.3-57.1)	28.5 (20.3-28.6)	28.6 (15.7-57.1)
**Pasta and whole cereal grains g/day**	79.3 (50.7-117.1)	75.0 (41.8-112.1)	79.3 (60.7-88.0)
**Eggs g/day**	14.0 (7.1-21.4)	14.1 (5.5-21.4)	8.9 (5.5-14.3)
**Plain yogurt g/day**	0.0 (0.0-0.0)	0.0 (0.0-9.4)	0.0 (0.0-0.0)
**Coffee g/day**	60.0 (17.1-90.0)	60.0 (15.5-90.0)	60.0 (20.3-90.0)

Data are expressed as mean±SD or median and interquartile range (IQR). Abbreviations are: MPF, Non-Processed or Minimally Processed Food. **p <0.05* vs Control Group.

Supplementary Table S9: Intake of Processed foods within the study population

	All patients<br>n=40	Control Group<br>n=20	CKD Group<br>n=20
**PF (PF+PCI) g/day**	451.2±241.5	431.7± 185.9	470.7±290.4
**% of tot food****	26.9 ±10.2	25.7 ±8.2	28.0±11.9
**PF *s' Subgroups***			
**Oil g/day**	40.0 (20.0-40.0)	40.0 (30.0-47.5)	35.0 (20.0-40.0)
**Olives g/day**	5.0 (1.4-10.0)	4.2 (0.0-10.0)	5.0 (3.5-9.2)
**Fats and dairy fats g/day**	0.0 (0.0-1.0)	0.0 (0.0-1.0)	1.0 (0.0-1.4)
**Concetrated sweeteners and sugar based products g/day**	11.2 (3.4-20.0)	9.3 (0.0-16.9)	13.5 (5.7-30.3)
**Bread Fresh g/day**	100.0 (50.0-200.0)	90.0 (50.0-187.5)	100.0 (50.0-200.0)
**Pizza artisanal g/day**	50.0 (40.0-50.0)	50.0 (50.0-50.0)	50.0 (38.7-50.0)
**Cheese g/day**	38.6 (21.9-62.6)	43.9 (19.3-68.2)	35.7 (24.4-61.8)
**Processed meat g/day**	14.3 (7.1-21.4)	14.3 (7.1-26.4)	14.3 (1.2-21.3)
**Canned Fish g/day**	7.1 (5.0-14.3)	7.1 (7.0-14.3)	6.1 (5.0-14.3)
**Smoked fish g/day**	0.0 (0.0-0.0)	7.1 (7.0-14.3)	7.1 (0.0-0.0)
**Canned Legumes g/day**	15.0 (0.0-31.1)	21.4 (2.5-42.6)	7.5 (0.0-21.4)
**Canned Vegetables g/day**	21.4 (0.0-42.8)	21.4 (0.0-42.6)	21.4 (16.5-42.8)
**Fruit juices g/day**	0.0 (0.0-0.0)	0.0 (0.0-0.0)	0.0 (0.0-0.0)
**Syrup-preserved fruit g/day**	0.0 (0.0-0.0)	0.0 (0.0-0.0)	0.0 (0.0-0.0)
**Dried fruit g/day**	0.0 (0.0-0.0)	0.0 (0.0-0.0)	0.0 (0.0-0.0)
**Added tree nuts g/day**	0.0 (0.0-0.7)	0.0 (0.0-3.0)	0.0 (0.0-0.0)
**Polenta g/day**	0.0 (0.0-0.0)	0.0 (0.0-0.0)	0.0 (0.0-0.0)
**Savory pies g/day**	0.0 (0.0-0.0)	0.0 (0.0-0.0)	0.0 (0.0-0.0)
**Gnocchi g/day**	0.0 (0.0-0.0)	0.0 (0.0-0.0)	0.0 (0.0-15.0)
**Artisanal biscuits and cakes g/day**	10.0 (0.0-10.0)	0.0 (0.0-10.0)	9.3 (0.0.12.2)
**Wine g/day**	0.0 (0.0-17.8)	0.0 (0.0-17.8)	0.0 (0.0-73.6)
**Beer g/day**	0.0 (0.0-33.0)	0.0 (0.0-8.2)	0.0 (0.0-33.0)
**Homemade sauces and gravies g/day**	5.0 (0.0-7.1)	1.7 (0.0-7.1)	5.0 (0.0-7.1)

Data are expressed as mean±SD or median and interquartile range (IQR). Abbreviations are: PF, Processed Food; PCI, Processed Culinary Ingredient. **p<0.05* vs Control Group.

Supplementary Table S10. Intake of Ultra-pocessed food within the study population

	**All patients** n=40	**Control Group** n=20	**CKD Group** n=20
**UPF g/day**	252.5 (154.0-300.6)	290.9 (184.4-413.4)	198.1 (124.8-282.6)*
**% of tot food**	14.4 (9.9-23.1)	18.8 (11.7-26.6)	12.3 (8.5-18.1)*
UPFs' Subgroups			
**Soft Drinks g/day**	33.0 (0-94.3)	28.3 (0-94.3)	0.00 (0-94.3)
**Alcoholics g/day**	0.0 (0.0-0.0)	0.0 (0.0-0.0)	0.0 (0.0-0.0)
**Packaged breads g/day**	0.8 (0.0-19.8)	5.0 (0-26.8)	0.0 (0.0-13.5)
**Buns g/day**	10.3 (0.0-30.0)	12.8 (0.0-30)	8.6 (0.5-30.0)
**Sweet and savoury snacks g/day**	14.3 (4.6-41.8)	19.2 (4.5 -62.8)	10.2 (0.0-18.1)
**Biscuits g/day**	10.7 (3.0-30.0)	17.1 (4.3-27.7)	8.6 (2.3-30.0)
**Ice-cream g/day**	0.0 (0.0-1.7)	0.0 (0.0-0.0)	0.0 (0.0-10.0)
**Chocolate g/day**	4.3 (0.0-12.8)	8.6 (0.0-14.5)	0.7 (0.0-8.6)
**Chips and French fries, g/day**	0.0 (0.0-14.3)	0.0 (0.0-26.3)	0.0 (0-15.0.0)
**Sausages and würstel g/day**	14.3 (14.3-30.7)	12.1 (10-18.6)	14.3 (10-21.0)
**Nuggets and sticks g/day**	10.0 (0-21.4)	10.0 (0-39.1)	12.1 (0-15.0)
**Fish sticks g/day**	0.0 (0.0-13.0)	10.0 (0.0-19.6)	0.0 (0.0-10.0)*
**Cereals and bars g/day**	0.0 (0.0-8.6)	3.6 (0.0-12.9)	0.0 (0.0-0.0)*
**Ultraprocessed dairy g/day**	0.0 (0.0-35.7)	15.2 (0.0-50.9)	0.0 (0.0-11.9)*
**Pre-packaged pizza, sandwich and savory pies g/day**	0.0 (0.0-0.0)	0.0 (0.0-0.0)	0.0 (0.0-0.0)
**Ready to eat sauces and gravies g/day**	2.3 (0.0-5.0)	1.8 (0.0-5.9)	3.9 (0.0-5.0)
**Ready to eat pasta gnocchi dishes g/day**	0.0 (0.0-15.0)	0.0 (0.0-0.0)	0.0 (0.0-15.0)
**Spreadable creams g/day**	0.3 (0.0-5.9)	2.6 (0.0-12.3)	0.0 (0.0-1.9)*
**Meat substitutes g/day**	0.0 (0.0-0.0)	0.0 (0.0-0.0)	0.0 (0.0-0.0)
**Pre-packaged vegetables g/day**	0.0 (0.0-0.0)	0.0 (0.0-0.0)	0.0 (0.0-0.0)
**Instant noodles or soups g/day**	0.0 (0.0-0.0)	0.0 (0.0-0.0)	0.0 (0.0-0.0)
**Candies g/day**	0.0 (0.0-3.7)	0.0 (0.0-1.2)	0.0 (0.0-8.9)
**Protein drinks g/day**	0.0 (0.0-0.0)	0.0 (0.0-0.0)	0.0 (0.0-0.0)*
**Margarine g/day**	0.0 (0.0-0.0)	0.0 (0.0-0.0)	0.0 (0.0-0.0)

Data are expressed as mean±SD or median and interquartile range (IQR). Abbreviations are: UPF, Ultra-Processed Food. *$p < 0.05$ vs Control Group .

Supplementary Table S11: Variation of dietary intake with advancing CKD in the study population

	**Control Group** n=20	**Stage 3 Group** n=6	**Stage 4 Group** n=9	**Stage 5 Group** n=5
**MPF g/day**	940.7±382.3	878.6 ±203.3	813.3 ±204.8	1036.1 ±206.1
**% of tot food**	54.1±13.3	60.8 ±12.2	53.0 ±17.6	61.9 ±13.5
**PF(PF+PCI)g/day**	431.7 ±185.9	452.3 ±168.3	503.0 ±375.1	434.6 ±281.7
**% of tot food**	25.7 ±8.2	30.5 ±10.1	28.3 ±13.5	24.4 ±12.8
**UPF g/day**	290.9 (184.4-413.4)	119.9 (63.7-190.5)*	201.8 (148.5-394.2)	266.4(136.3-287.7)
**% of tot food**	18.8 (11.7-26.6)	8.5 (5.0-11.8)*	12.7 (10.9-27.6)	14.4 (7.5-19.2)

Data are expressed as mean±SD or median and interquartile range (IQR). Abbreviations are: MPF, Non-Processed or Minimally Processed Food; PF, Processed Food; PCI, Processed Culinary Ingredien; UPF, Ultra-Processed Food. **p <0.05* vs Control Group
